# Effects of Chemical Cleaning on the Ageing of Polyvinylidene Fluoride Microfiltration and Ultrafiltration Membranes Fouled with Organic and Inorganic Matter

**DOI:** 10.3390/membranes12030280

**Published:** 2022-02-28

**Authors:** Mariny Chheang, Narapong Hongprasith, Chalita Ratanatawanate, Jenyuk Lohwacharin

**Affiliations:** 1 Department of Environmental Engineering, Faculty of Engineering, Chulalongkorn University, Bangkok 10330, Thailand; marinychheang2@gmail.com (M.C.); narapong.h@chula.ac.th (N.H.); 2 Environmental Nanotechnology Research Team (ENV), National Nanotechnology Center, Pathumthani 12120, Thailand; chalita@nanotec.or.th; 3 Research Network of NANOTEC-CU (RNN) on Environment, Department of Environmental Engineering, Faculty of Engineering, Chulalongkorn University, Bangkok 10330, Thailand

**Keywords:** sodium hydroxide, tannic acid, membrane fouling, hydrolysis, lifetime

## Abstract

Herein, the effects of cleaning with sodium hydroxide and citric acid solutions as cleaning reagents on the changes in the properties of two hollow-fiber PVDF microfiltration (MF) and ultrafiltration (UF) membranes fouled with organic and inorganic matter were investigated. Accelerated membrane ageing was induced by use of high concentrations of tannic acid and iron oxide (Fe_2_O_3_) particles in the feed water; these conditions were kept with different membrane soaking times to observe temporal effects. It was found that tannic acid molecules adsorb onto the membrane surface that results in changes in surface characteristics, particularly surface functional groups that are responsible for enhancing membrane’s hydrophilicity. Experimental results demonstrate that NaOH had a stronger effect on the tensile strength and surface chemistry of the fouled MF and UF membranes than citric acid. Prediction of lifetime by an exponential (decay) model confirmed that the UF membrane cleaned with NaOH would be aged within about 1.8 years and the MF membrane after about 5 years, at cleaning every 15 days, downtime 2 h per cleaning, when a 10% tensile strength decrease against the original membrane is allowed.

## 1. Introduction

The processing of agricultural products produces processed waters with different characteristics that depend on a variety of raw materials, crop growing locations, technological mechanization, and the availability of a secure energy supply. In cashew nut processing, either the roasting process or the steam cooking process is widely used. In the former process, processed water is generated during a quenching step of the roasted cashew nuts. In the boiling process, on the other hand, the water is drained from the pressure cooker. Cashew nut shell liquid (CNSL) contains recalcitrant organic compounds that are not easily biodegradable, such as long-chain phenolic compounds [1]. Direct disposal of CNSL can cause irreparable damage to ecosystems and threaten aquatic and terrestrial fauna and flora.

The microfiltration (MF) and ultrafiltration (UF) processes ensure the removals of bacteria, germs, viruses, and colloidal particles from processed waters. In addition, quality of the treated water remains the same, independent of pollution level of the feed water. Nonetheless, the MF and UF processes have relatively low efficacy on organics and ions removal, as compared to nanofiltration and reverse osmosis. Different integration of these pressure driven membrane processes have been applied in a variety of wastewater treatment settings: particularly MF and UF as pretreatment to other unit processes [2]. Compared to the alternatives of electrodialysis and reverse electrodialysis—which are promising desalination technologies that can transform salinity gradient energy into electricity [2,3,4]—electrodialysis shows a relatively higher total organic carbon rejection than nanofiltration [3] but it is still prone to organic and inorganic fouling. Meanwhile, pretreatment with coagulation can enhance organic removal efficacy by the UF process [2].

Recent studies have reported the use of lab-prepared polymeric membranes, made of cellulose acetate and polyethylene glycol, in removing iron and zinc ions from synthetic wastewaters through an electrodialysis process [5,6]. The authors found that membrane enrichment with chitosan-silver ions increased electrical conductivity and hydrophilicity [5], while adding SiO_2_ nanoparticles to the polyvinyl alcohol matrix improved proton conductivity [6]. As for commercially available polymeric MF and UF membranes, hollow-fiber membrane modules are widely used for water treatment and reuse due to their high packing density and relatively low manufacturing cost [2]. Hollow-fiber membranes are usually pressurized in a housing or immerged in a basin and are mainly made of polyvinylidene fluoride (PVDF) or polyethersulfone (PES) [7]. PVDF is widely used as a polymeric membrane material due to its outstanding properties: i.e., high mechanical strength, flexibility, thermal stability, and chemical resistance [8].

Membrane fouling is a major impediment to the long-term operation of small water treatment plants such as CNSL plants. It causes a decrease in permeate flux, a rapid increase in transmembrane pressure (TMP), and probable degradation of the mechanical properties of the membranes [2,7]. Fouling by organic compounds, known as “organic fouling”, may require extensive cleaning with chemicals such as chlorine or a strong alkali to restore membrane permeability. This is because organic molecules can adsorb on the inner walls of membrane pores and cause membrane pore plugging that cannot be effectively removed by physical cleaning [2]. In electrodialysis processes, the removal of anionic organic and inorganic foulants from ion exchange membranes can also be performed through “cleaning-in-place” methods using acidic and alkaline solutions [3].

Unfortunately, chemical cleaning can not only remove contaminants, but also attack the membrane materials, resulting in polymer degradation. Chemical cleaning has a relatively greater impact on the chemical and physical properties of polymeric membranes than physical cleaning. Polymers are broken down into monomers during hydrolysis [9], a reaction that involves the consumption of a water molecule while breaking the covalent bond that holds two components of a polymer together (such as an ionized and an unionized component). Various studies have confirmed that sodium hydroxide and citric acid, which are commonly used membrane cleaning solutions, affect the properties and performance of membranes. These chemicals create a net negative charge on the membrane surface, which increases hydrophilicity and leads to a decrease in membrane permeability [10,11]. Elevated temperatures, bleach solution concentration, and extreme pH environments can exacerbate polymer degradation [12].

Increasing attention is being paid to the integrity of membranes, as membrane damage will result in deterioration of permeate water quality. In the case of hollow fiber membranes, previous studies have focused on their mechanical properties, which are directly related to a faulty structural arrangement of the membrane module or the external pressure exerted on the fibers during operation [7]. On the other hand, chemical attack (or oxidation) leading to membrane fiber failure may be caused by incompatibility between chemicals in the feedwater with the membrane material. Ravereau et al. [8] studied the degradation of two PVDF hollow fiber membranes in chlorine solutions with different pH and found that prolonged exposure of PVDF membranes to chlorine leads to the formation of double bonds, chain scission, and crosslinking, especially in acidic solutions. In addition, strong reactivity of non-fluorinated aliphatic or cycloaliphatic additives with bleach accelerates chain scission.

Currently, a study examining the effects of fouling by CNSL components (i.e., phenolic compounds and iron oxide particles) on membrane integrity is still pending. A recent study in full-scale water treatment plants revealed that aged membranes may be more susceptible to the loss of mechanical properties than newer membranes [13], and yet the relationship between the fouling effects and changes in surface functional groups associated with chemical cleaning has not yet been studied in detail. Unfortunately, most existing studies on membrane ageing by chemical treatments only focus on new membranes. It is to our best knowledge that this current study is the first to investigate the effects of sodium hydroxide and citric acid solutions on the physico-chemical properties of PVDF MF and UF membranes fouled by simulated CNSL. Since the degree of polymer damage is time-dependent, we intend for this paper to establish better understanding of the mechanism of membrane degradation located on the additives and backbone structures of the membranes resulting in extended chemical attacks when exposure duration is varied. The main objective of this study is to establish a relationship between cleaning procedures and changes to the mechanical and physicochemical properties of two PVDF membranes, as well as to establish the lifetimes of the two membranes as affected by cleaning procedures.

## 2. Materials and Methods

### 2.1. Hydrophilized PVDF Hollow-Fiber MF and UF Membranes

An outside-in PVDF hollow-fiber MF membrane (model no. LE-US02-125, Kuraray Co., Ltd., Tokyo, Japan) and UF membrane obtained from National Nanotechnology Center (NANOTEC, Pathumthani, Thailand) were used in this study. These membranes were packed in a cartridge-like configuration and designed for use in a variety of applications, e.g., water reuse. The nominal pore diameters of the MF and UF membranes are 0.02 μm and approximately 15 nm, respectively (molecular-weight cut-off (MWCO) of 100–200 kDa). The pressurized module has an effective filtration area of 0.0864 m^2^ (effective length of 27 cm, 102 fibers) and operating flux of 75–100 L/m^2^-h for MF at a transmembrane pressure (TMP) of <0.1 MPa (or 1 bar). The effective filtration area of the UF membrane is 0.0143 m^2^ with 14 fibers that can be used at a TMP < 0.2 MPa, giving a filtrate flux of 50–70 L/m^2^-h.

**Figure A1:**
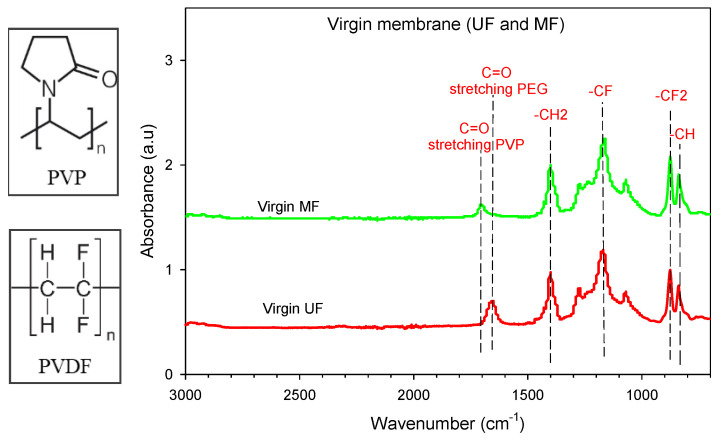
FTIR spectra of virgin MF and UF membranes.

**Figure A2:**
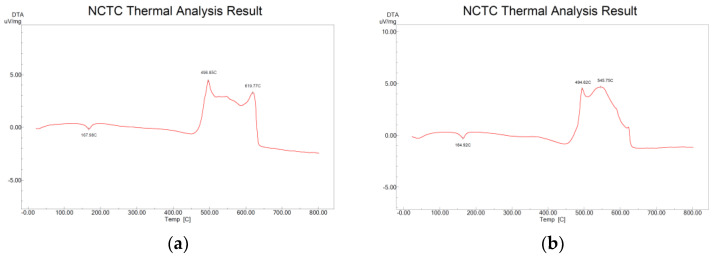
DTA analysis of (**a**) virgin MF and (**b**) virgin UF membranes at the nitrogen gas flow rate of 50 mL/min.

**Figure A3:**
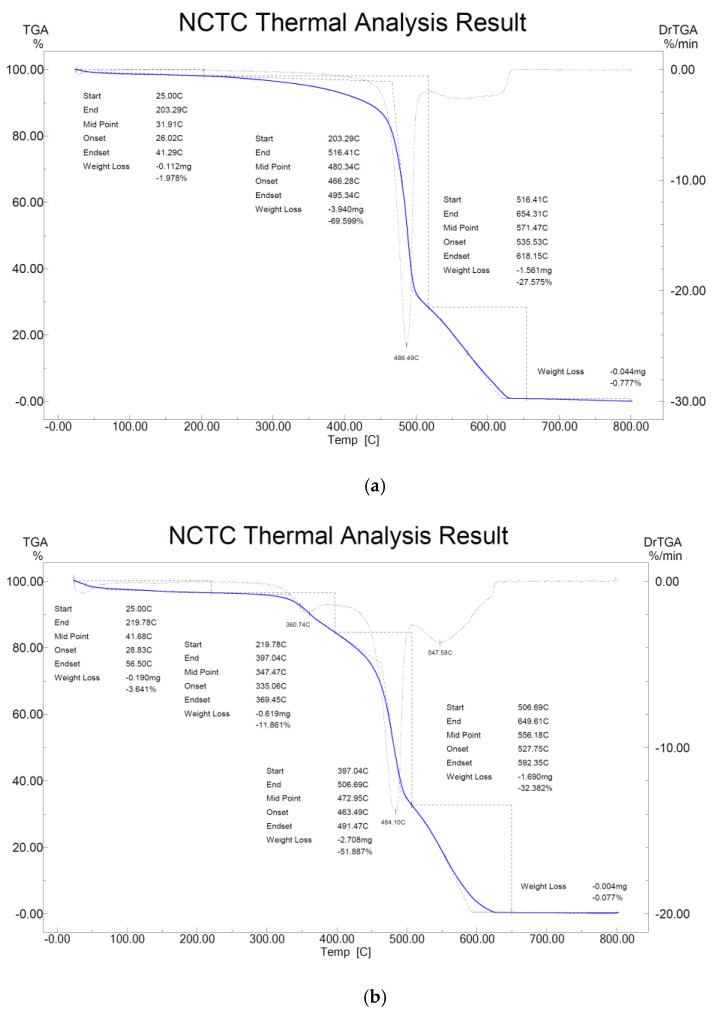
TGA analysis of (**a**) virgin MF and (**b**) virgin UF membranes at the nitrogen gas flow rate of 50 mL/min.

The properties of the UF and MF membranes are summarized in Table A1 (Appendix A). The functional groups and chemical structures of the UF and MF membranes analyzed by FTIR spectroscopy confirm characteristic peaks of PVDF (e.g., –CF and –CF2 peaks) and additives (i.e., Polyvinylpyrrolidone (PVP) for MF and Polyethylene glycol (PEG) for UF) as shown in Figure A1. Thermal gravimetric analysis (TGA) of the PVDF membranes showed that the PVDF content of MF was 69% and UF was 64% based on the weight loss of PVDF decomposition at elevated temperatures of 385–510 °C (Figure A2 and Figure A3).

### 2.2. Synthetic Wastewater

The cashew nut processing industry typically produces processed water containing high concentrations of organic matter, usually refractory, and iron oxide [1]. To reflect this, two synthetic feedwaters were prepared: Water A, which represents organically contaminated water; and Water B, which represents organic–inorganic contaminated water to which iron oxide (Fe_2_O_3_) was added. Both waters contained tannic acid (Wako Pure Chemical Industries, Ltd., Osaka, Japan) as a surrogate for organics in the cashew nut processing wastewater at a relatively higher concentration than would be expected in real wastewater to accelerate membrane fouling. Iron oxide particles (CAS No. 458700010, Iron (III) oxide, 95% pure, Acros Organics, NJ, USA) were used as model iron oxide in the processed water. The characteristics of the synthetic cashew nut waters used in this study are shown in Table 1. Based on the laser scattering particle size distribution analyzer (LA -960, Horiba), the mean size of iron oxide particles dispersed in MilliQ water and tannic acid solution were 3.2 and 13.84 μm, respectively.

### 2.3. Membrane Filtration Processes

**Figure 1:**
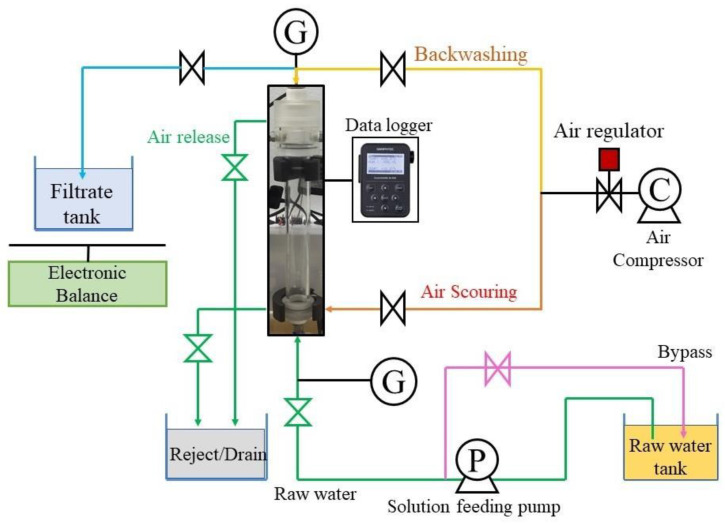
Schematic diagram of membrane filtration experiments. Notations: C denotes air compressor; G denotes digital pressure gauge; and P denotes pump. (Modified from “Operation Procedures for Laboratory Outside-in Filtration Test”, obtained via private communication with Kuraray Co., Ltd., Tokyo, Japan).

**Figure A4:**
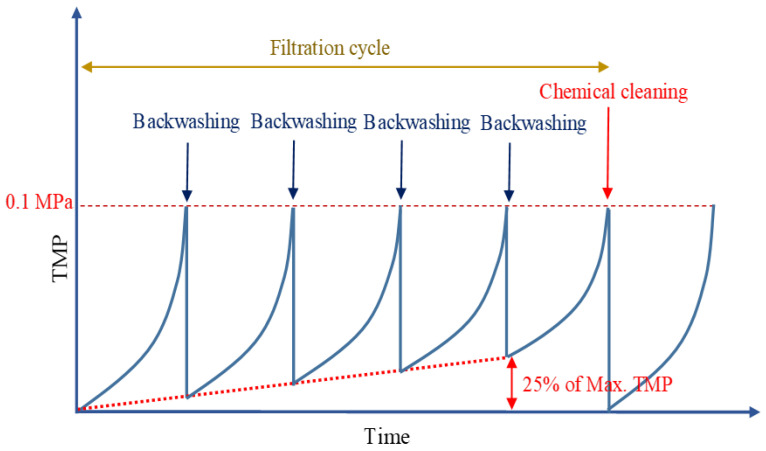
Transmembrane pressure graph.

The bench-scale MF and UF system was operated with dead-end filtration under an outside-in mode (Figure 1). Briefly, the filtration cycle comprised of filtration, air scouring, physical cleaning/chemical cleaning, drainage, and refill. During filtration, membrane fouling would lead to a gradual increase in TMP. The membranes were subjected to physical cleaning when the TMP reached nearly 100 kPa. Physical cleaning comprised of several steps as follows: first, pressurized air at 200 kPa was supplied from the filtrate side for 10 s; next, pressurized air was supplied from the lower end of the membrane housing to exfoliate particles adhered to the membrane surface for 60 s; finally, the solution (containing detached particles) inside the membrane housing was drained out. Inevitably, after numerous filtration cycles, physical cleaning became inefficient at removing fouling, at which point the fouling was termed as “irremovable fouling”. At this point, the initial TMP of the next filtration cycle, even immediately after backwashing, was approximately 25 kPa (~25% of the maximum TMP) due to the accumulation of non-removable impurities; to resolve this, cleaning with reagents was performed. During the filtration experiments with either one of the two prepared feed waters, TMP trends and permeate water flow rates were recorded. A summary of the filtration procedure is shown in Figure A4. The total filtration volume during MF-membrane filtration of feed water A (tannic acid only) was 2546 L/m^2^, and of feed water B (tannic acid with iron oxide) was 3241 L/m^2^.

### 2.4. Chemical Cleaning Regime

Chemical cleaning is usually used to remove contaminants that are strongly adsorbed on membrane surfaces and cannot be removed by physical cleaning alone. The performance of chemical cleaning must be evaluated not only by its ability to restore membrane flux, but also by its ability to maintain product water quality, which depends on several factors, including the concentration of the cleaning agents, temperature, cleaning time, and hydrodynamic conditions [2,8]. In this study, sodium hydroxide (NaOH) and citric acid (CA) were used as membrane cleaning agents.

Amounts of 2.5 wt.% NaOH and 2.0 wt.% citric acid were used to clean the membrane fouled by tannic acid and deposits of multivalent cations and metal oxides, respectively, at 25 °C. The selected concentrations of NaOH and citric acid were in accordance with the usual concentrations used for chemical cleaning in real pilot and large-scale wastewater treatment plants. The alkaline cleaning conditions allowed for hydrolysis of organic compounds such as polysaccharides and proteins. The cleaning time was extended in order to accelerate the ageing of the membrane. Therefore, the membrane lifetime was tested by cutting the membranes (UF and MF membranes) into small segments, about 10 cm long, from which 2 or more fiber samples were taken and immersed in a beaker containing NaOH or citric acid for 30 min to 2 weeks.

### 2.5. Membrane Characterization

**Figure A5:**
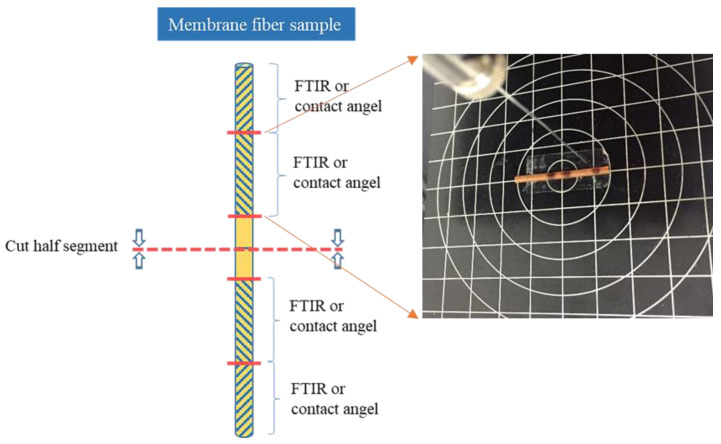
FTIR and contact angle analyses of representative specimens of virgin and fouled MF and UF membranes.

Attenuated Total Reflectance Fourier Transmission Infrared (ATR-FTIR) spectroscopy, used to follow the changes in the organic and inorganic functional groups of the membrane, was analyzed with a scan resolution of 4 cm^−1^ from a wavenumber of 6000 to 400 cm^−1^ using the Thermo Scientific Nicolet 6700 FTIR (Waltham, MA, USA) spectrometer. The contact angle was analyzed using an OCA 15 Plus (DataPhysics Instruments GmbH, Filderstadt, Germany) by Pico droplet measurement and dispensing a 0.5 µL droplet of water. To obtain representative values for the entire membrane sample of virgin and chemically cleaned membranes, at least three points on the membrane surface were randomly selected to perform the ATR-FTIR and contact angle measurements (Figure A5). If the resultant membrane contact angle value was less than 50°, it was considered hydrophilic, and any higher values were considered hydrophobic [14,15].

Tensile tests were performed to determine the tensile strength and elongation (maximum elongation without permanent deformation) of MF and UF membranes according to ASTM D882 standard. All membrane samples (12–13 cm long) were rinsed and immersed in distilled water for approximately 1 h to remove any detergent residue remaining on the membrane surface. The membrane fibers were dried overnight in a desiccator and stored in a stainless-steel box until analysis. Thermogravimetric/differential thermal analysis (TG and DTA) was performed by measuring the thermal stability of the membrane samples with a DTG-60AH analyzer (Shimadzu Corp., Kyoto, Japan) with a heating rate of 10 °C/min from room temperature to 800 °C under a nitrogen gas flow of 50 mL/min.

### 2.6. Lifetime Prediction

The Weibull distribution was used as the lifetime model because it is robust and can be applied to many types of lifetime data and has only two model parameters (i.e., β: shape parameter; and η: scale parameter) [16]. The probability density function (PDF) and the cumulative distribution function (CDF) are important statistical functions used to describe a lifetime distribution. Two parameters of the Weibull PDF are defined as follows [17]:

(1) $$f\left(t\right)=\frac{\beta }{\eta }{\left(\frac{t}{\eta }\right)}^{\beta -1}\;e^{-{\left(\frac{t}{\eta }\right)}^{\beta }},\;\left(\mathrm{for}\;t\geq 0\right)$$

where *β* is the shape parameter; *η* is the scale parameter or characteristic life.

The reliability function of Weibull, *R*(*t*) distribution is described as follows:

(2) $$R\left(t\right)=e^{-{\left(\frac{t}{\eta }\right)}^{\beta }}$$

The Weibull failure rate function or hazard function, *λ*(*t*) can be described as follows:

(3) $$\lambda \left(t\right)=\frac{f\left(t\right)}{R\left(t\right)}=\frac{\beta }{\eta }{\left(\frac{t}{\eta }\right)}^{\beta -1}$$

When

- populations with β < 1 exhibit a failure rate that decreases with time,
- populations with β = 1 have a constant failure rate, and
- populations with β > 1 have a failure rate that increases with time.

**Figure A6:**
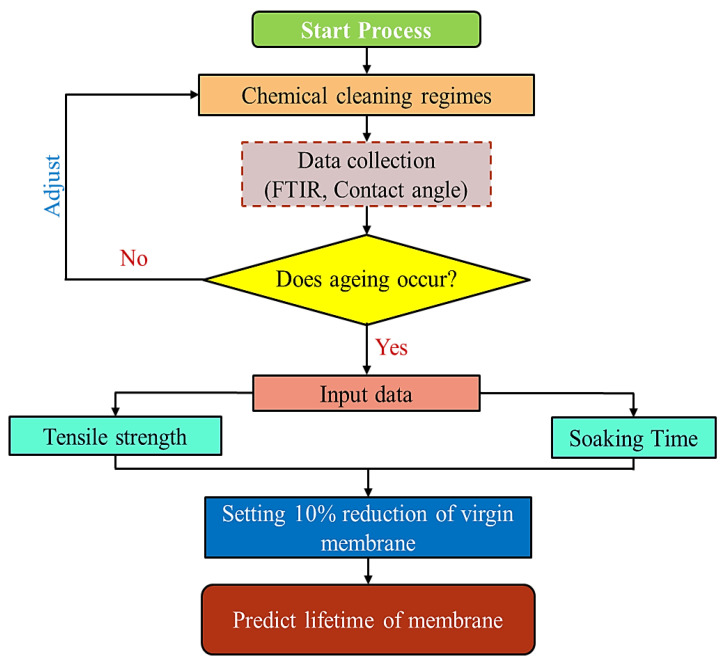
Flow diagram of membrane lifetime prediction.

The procedure for predicting the lifetime of the hollow fiber membranes (UF and MF) after chemical cleaning at different soaking times is described in Figure A6. Briefly, the measured physical properties, i.e., tensile strength and soaking time, of the membranes were the input parameters for Equation (3), which were used to simulate the lifetime. According to Arkhangelsky et al. [18], the tensile strength decreased by 5% to 30% after ageing, while Le-Clech [19] reported that the breaking stress of the hollow fibers decreased by more than 0.004%, which is a significant change between virgin and aged membrane. The criteria for assessing whether the membrane had aged included a combination of tensile strength, effluent quality, and FTIR results. Therefore, to predict the life of the membrane, the tensile strength was assumed to decrease to 10% of the tensile strength of the unaged membrane due to ageing. In addition, the ATR-FTIR result helps us to confirm the ageing of the polymeric membranes as this can be seen from the shift in the characteristic peaks of PVDF and additives.

## 3. Results and Discussion

### 3.1. Membrane Filtration Performance

The MF and UF membranes were filtered with two different types of feed water containing TA and iron oxide, which are important organic and inorganic constituents that cause membrane fouling, to simulate the low-pressure filtration treatment of processed water from the cashew nut processing industry. Membrane fouling can be observed as an increase in TMP or a decrease in permeate flux and permeate water quality. Our previous study indicates that ultrafiltration of the aqueous suspension of iron oxide particles might suffer from pore blocking and cake fouling [20]. In addition, iron oxide particles could adsorb organic matter via ligand exchange and electrostatic interaction [21].

**Figure A7:**
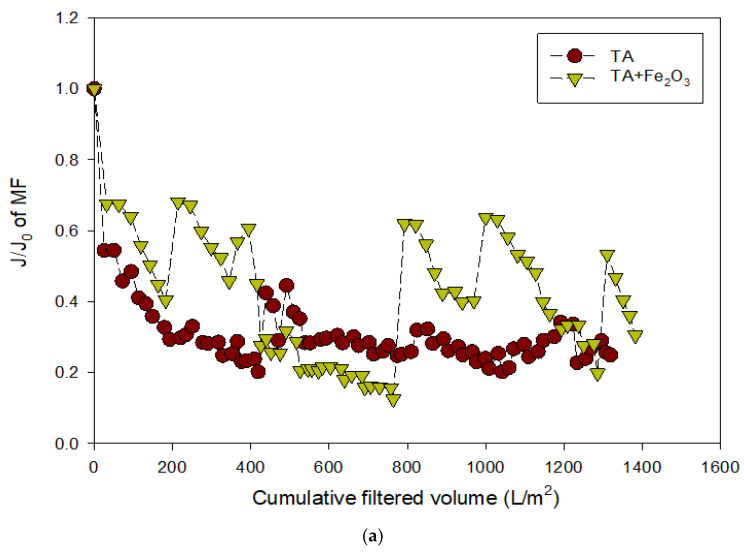

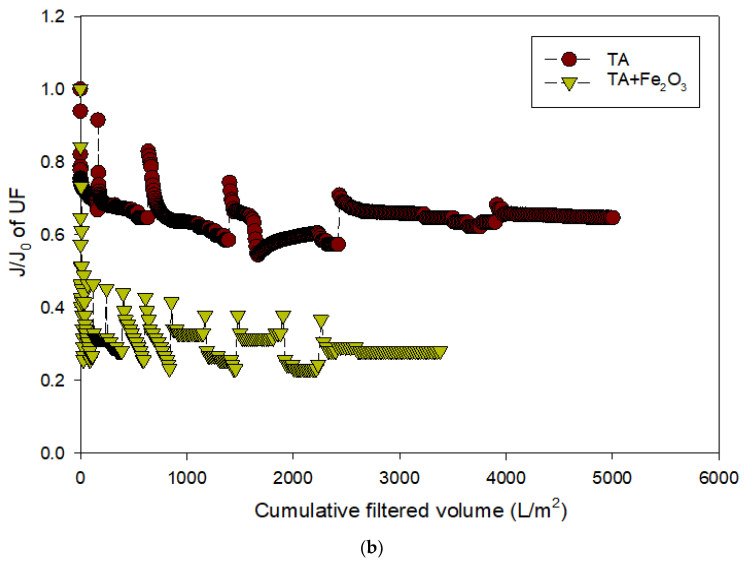
Flux decline at filtering tannic acid and tannic acid + Fe_2_O_3_ feed waters: (**a**) MF and (**b**) UF membranes. Effluent permeate flux (J) normalized to initial permeate flux (J_0_).

The performance of MF membrane evaluated on flux decline behavior shows that tannic acid with iron oxide (TA + Fe_2_O_3_) and tannic acid (TA) alone fouled the membrane significantly. J/J_0_ decreased to about 0.3 at a filtered volume of 200 L/m^2^ of the TA-containing water (Figure A7), which corresponds to a COD loading of 0.8 kg-COD/m^2^. It is worth noting that iron oxide particles were completely retained as their particle size was larger than the pore diameter of the MF membrane (~160 to 700 times larger). In the presence of iron oxide, the flux of the membrane was restored to some extent after backwashing. However, when tannic acid was filtered alone, no recovery of flux was observed. The removal efficiency of total organic carbon (TOC) was about 5% of the original TOC in the feed water for feed water A and about 6% for feed water B (Table A2), indicating that a small amount of retained organic molecules causes severe fouling of the MF membrane. The small difference between the two feed waters indicates that the presence of iron oxide particles did not affect the removal of organics.

**Figure 2:**
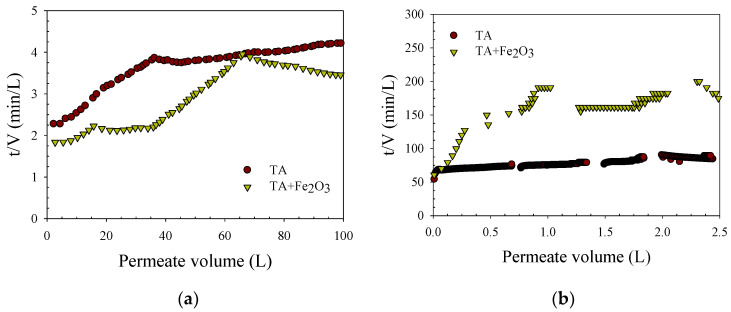
Reverse cumulative flux (t/V) versus permeate volume (L) at filtering tannic acid (TA) and TA + Fe_2_O_3_ feed waters (**a**) MF and (**b**) UF membranes. Initial TA: 100 mg/L; initial Fe_2_O_3_: 10 mg/L; and solution pH: 7.5.

The previous study shows that fouling depends on hydrodynamic conditions and foulant properties [2,20,22]. The cake filtration was distinguished from other fouling mechanisms by plotting t/V versus V (see Figure 2), where a linear relationship identifies cake filtration as the dominant fouling mechanism [22,23]. TA filtration with the MF membrane shows a curve from the initial phase up to about 50 L filtrate volume, indicating pore-clogging processes by low molecular weight TA. In the presence of iron oxide (TA + Fe_2_O_3_), t/V was linearly correlated with V from a filtrate volume of about 35 L, indicating that the fouling mode shifted to cake filtration. Thereafter, a decrease in t/V with increasing V was observed, probably caused by air scouring during filtration, which peeled off the loose cake of iron oxide.

In contrast to the MF membrane, the UF membrane exhibited a more rapid and steeper flux decrease in the presence of iron oxide particles (feed water B) compared to the filtration of tannic acid alone (Figure A7). This was also confirmed by the steep increase in the plot of t/V versus V for the UF membrane in Figure 2b. Previous studies reported that coating membrane surfaces with iron oxide particles led to a reduction in fouling [24]. This discrepancy in membrane fouling by iron oxide could be due to the different relative sizes of the iron oxide particles in their study.

**Figure 3:**
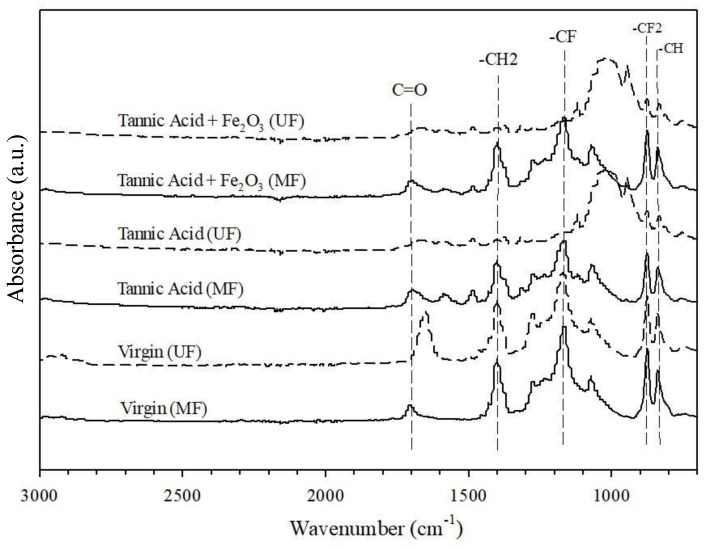
FTIR spectra of virgin and fouled UF and MF membranes filtrated with TA and TA + Fe_2_O_3_ feed waters.

ATR-FTIR spectroscopy was used to study the chemical properties of the fouled membranes, as shown in Figure 3. The characteristic peaks of the virgin UF membranes at wavenumbers of 1180 cm^−1^ [25], 1234 cm^−1^ [26], and 1275 cm^−1^ [27], which represent the -CF out-of-plane stretching [28], disappeared from the spectra of the TA-filtered and TA -Fe_2_O_3_-filtered UF membranes. This phenomenon was not observed for MF membranes, suggesting that organic impurities cover the surface of the UF membrane more than that of the MF membrane. This was also confirmed by the appearance of a peak at 1000–1100 cm^−1^ for only the UF membranes. In addition, the carbonyl group (i.e., C=O), which may be derived from a hydrophilic additive of PVP, was observed on both virgin and fouled MF membranes. The carbonyl group, which is derived from PEG, and the methylene group (i.e., -CH2) on virgin UF membrane disappeared from the spectra of the fouled UF membrane (Figure 3), suggesting surface coverage by fouling materials.

Overall, both membranes were able to adequately retain iron oxide particles, but they did not have a strong effect on the chemical properties of the membranes. Tannic acid, on the other hand, was an important influencing factor that could change the chemical properties of the membrane by adsorption on the membrane surface; although, only a small amount was removed. This aligns with our previous study, which found that tannic acid coating on PVDF membranes reduced the membrane’s water contact angle by 17–27%, depending on the acid’s molar concentration [15].

### 3.2. Chemical Cleaning and Effects on Membrane Properties

#### 3.2.1. NaOH and Citric Acid Cleaning

**Figure 4:**
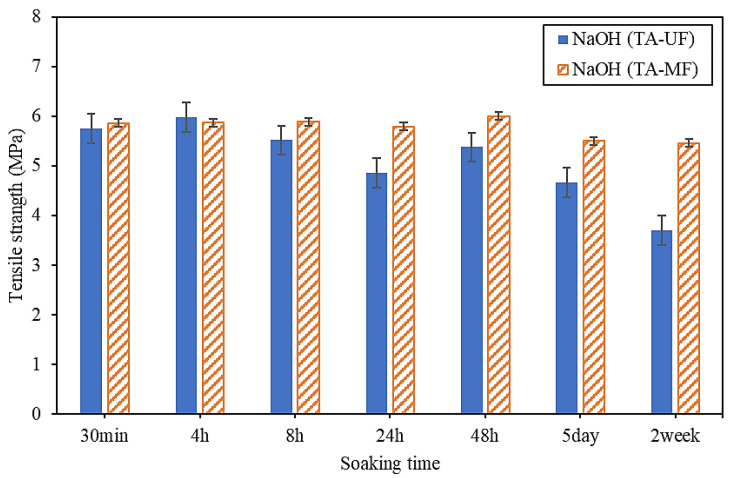
Tensile strength of MF and UF membranes filtrated with tannic acid in NaOH solutions.

Cleaning the membranes with 2.5% wt NaOH affected the physical and chemical properties of the PVDF hollow fiber membrane. Physicochemical properties that were considered in the cleaning and ageing studies included contact angle and chemical functional groups of the membrane surface [13,29], whereas the physical property considered was the commonly referred to parameter of tensile strength. Changes to the membrane’s tensile strength with increasing cumulative cleaning time are shown in Figure 4. The tensile strength of the UF membrane obviously decreased with longer durations of cleaning with NaOH, indicating that the mechanical strength of the membranes deteriorated with increasing cleaning time. The membrane surface’s hydrophobicity after intensive cleaning at different exposure times was evaluated by contact angle measurements. The fouled MF and UF membranes after filtration of TA solution then cleaned with NaOH showed a gradual decrease in contact angle up to a soaking time of 5 days (Figure A7). Subsequently, the contact angle increased significantly after a soaking time of 2 weeks, indicating that prolonged exposure to NaOH changed the chemical structure of the membrane. Caustic cleaning agents have been reported to lead to dehydrofluorination phenomena, in which C=C bonds are formed when the H-F units are eliminated from the polymer [8,19].

FTIR analysis characterized the fouling materials deposited on the membrane surface after filtration (Figure 3). The peak at 1700 cm^−1^ belongs to the carbonyl group (C=O), associated with the PVP additive commonly added to increase the hydrophilicity of the membrane [25]. This characteristic peak of the carbonyl group (C=O) disappeared from the IR spectra of the membrane after cleaning with NaOH for 2 weeks. There were peaks representing CH_2_–OH deformation and C=C bonding of the fouled membrane after filtration of TA solution. After soaking in NaOH from 30 min to 5 days, the CH_2_–OH and C=C peaks gradually decreased and disappeared after a soaking period of 2 weeks, indicating that the cleaning with NaOH must be sufficiently long to achieve complete removal of organic contaminants.

**Figure A8:**
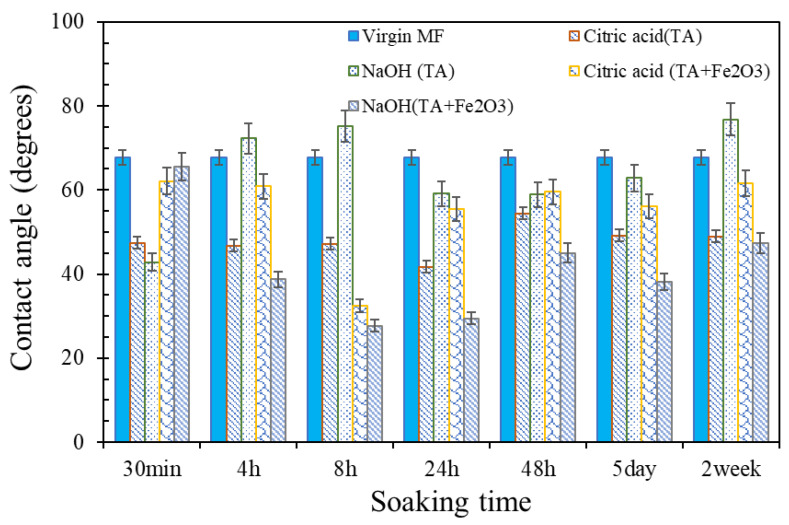
Contact angle of fouled MF membranes cleaned with NaOH and Citric acid of both types of wastewaters.

Citric acid is normally used to remove inorganic particles (e.g., iron oxide) and debris from the membrane surface. Analysis of the contact angle helps us to understand that the MF and UF membranes tend to have lower contact angles after cleaning with citric acid (Figure A8) than the virgin membranes. This indicates a decrease in hydrophobicity and shows an opposite trend to that observed when the membranes were cleaned with NaOH.

#### 3.2.2. Polymer Hydrolysis and Ageing

**Figure 5:**
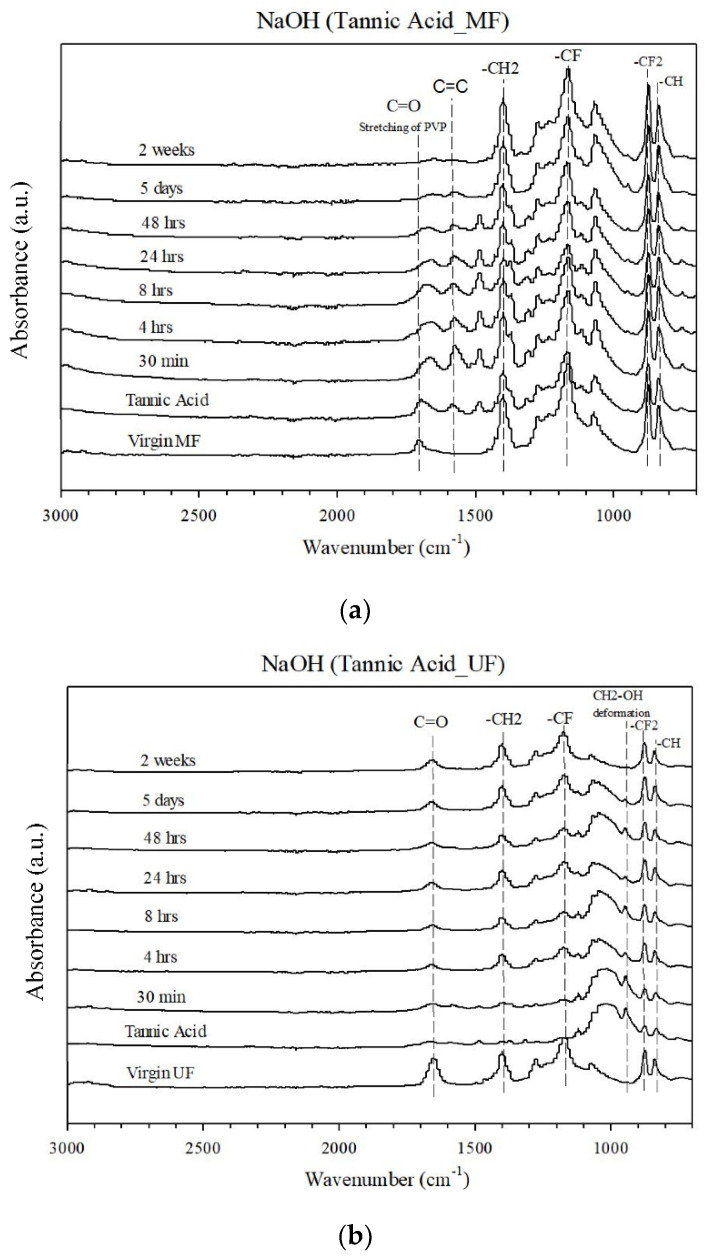
FTIR spectra of virgin and fouled (**a**) MF and (**b**) UF membranes filtrated with the tannic acid solution after soaking in NaOH at different periods from 30 min to 2 weeks.

To combat the hydraulically irremovable fouling that occurs after long-term filtration, NaOH and citric acid were used to clean the fouled membranes. A previous study indicated that the support material of commercial UF membranes made of polyethylene terephthalate broke down into their monomers under strong alkaline conditions through the hydrolysis reaction [30]. The results of FTIR and contact angle measurements (Figure 5 and Figure A8) indicated that the membrane cleaned with NaOH became increasingly hydrophobic after extensive cleaning with NaOH. It is known that PVP was added to PVDF to improve the hydrophilic property of the MF membrane, and the extensive cleaning with NaOH probably caused a loss of the hydrophilic additive since it was previously found that soaking the fouled membrane in NaOH for a long period of time can result in a crosslinking reaction on the membrane surface, causing the membrane surface to become hydrophobic [31]. Similarly, hydrolysis of the membrane may have occurred as evident from the loss of the characteristic C=O bond absorbance band in the 2-week-soaked membrane (see Figure 5). This is consistent with the study of Hashim et al. [32], which found that by soaking virgin PVDF hollow-fiber membranes in NaOH solutions, a reaction between NaOH and PVDF was initiated even with low NaOH concentrations and was aggravated at the 24-hour treatment time. Other studies have concluded that—based on the FTIR results—additives in PVDF membranes degraded during hypochlorite soaking [19,29]. Back to the results of our study, the hollow fiber membrane’s tensile strength changed slightly after hydrolysis, while elongation decreased by 23%, as shown in Table 2. Similar results have also been reported by a previous study where commercial PVDF hollow-fiber membranes experienced changes to their elongation between 84% and 183%, with moderate elongation reduction occurring when the membranes were treated with 1% and 4%wt NaOH solutions [32]. In the same study, the authors also reported a loss of mechanical integrity in the membrane after treatment with 10%wt NaOH.

Moreover, Young’s modulus is a measure of the stiffness of the material or its resistance to elastic loading. The modulus of elasticity of PVDF is generally 145,000–333,500 psi [33]. It is generally assumed that the mechanical property of the membrane is not important because it is held in place by a support material. This is not the case with the hollow fiber membrane because it is self-supporting, so its mechanical strength becomes very important [8,19]. For example, fibers with a high modulus of elasticity can easily withstand higher operating pressures.

### 3.3. Lifetime Estimation

Membrane ageing is complex [8,13]. A previous study has suggested that cleaning rate is not limited to characteristic changes, and there are relatively few links to the fouling rate [34]. In this study, accelerated membrane ageing was induced by use of high concentrations of tannic acid and iron oxide particles in the feed water; these conditions were kept with different membrane soaking times in order to observe temporal effects. In accelerated ageing tests, the fibers need a longer soaking time to fail. The fouled membrane was soaked in NaOH 2.5 wt.% and citric acid 2 wt.% at different cumulative soaking times from 30 min to 2 weeks with seven samples. NaOH was chosen to predict the ageing of the membrane because FTIR and contact angle results showed that NaOH causes hydrolysis of the membrane by a strong base and changes the structure of the membrane after prolonged soaking. The membrane filtered with a tannic acid solution was chosen to determine the ageing of the membrane and the NaOH solution was used for removing organic contaminants from the fouled membrane.

The two most important ageing factors were the chemical properties of the membranes (analyzed by FTIR and contact angle) and the physical properties of the membranes (i.e., tensile strength and elongation). The tensile strength data were used to analyze the ageing of the membranes using the Weibull model. The tensile strength data were fitted to an exponential model, which is also part of the Weibull distribution. To predict the time of membrane ageing, two different scenarios were created: a minimum tensile strength decrease of 10% and a maximum of 30% against the virgin membrane were assumed as the definitions of when the membrane had deteriorated and ageing had occurred [19]. The membrane ageing results based on the exponential equation are shown in Table 3. According to Judd [35], membranes should typically be cleaned two times per month (every 15 days) with chemical reagents, with each cleaning taking 2 h (2 h downtime).

**Figure 6:**
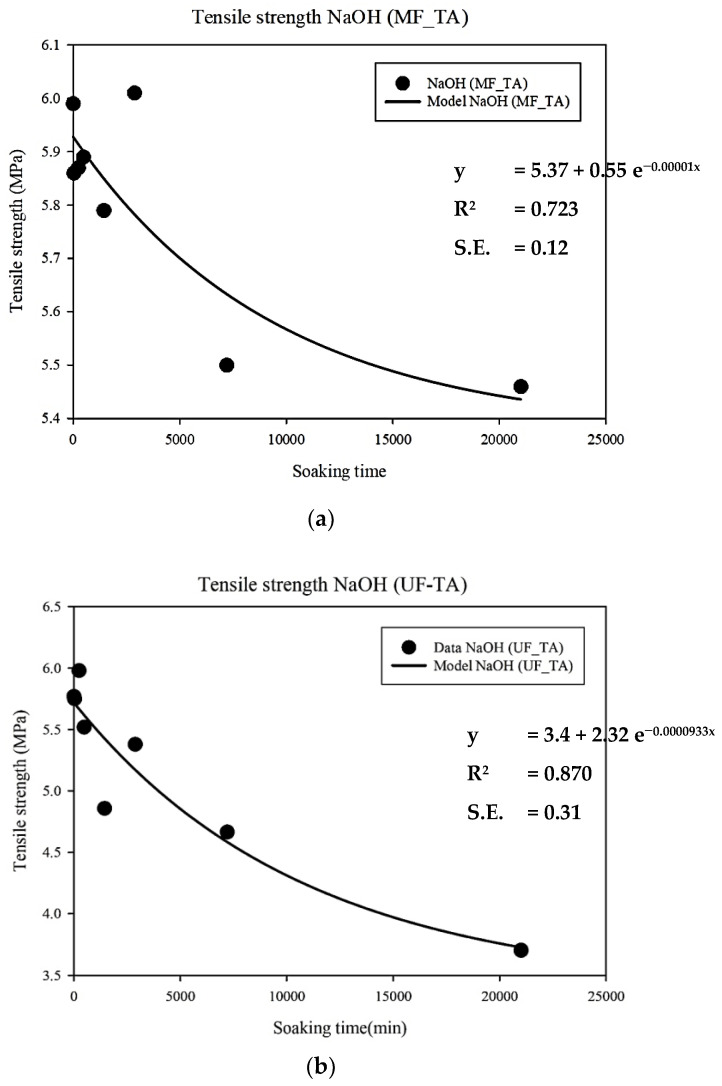
Tensile strength of (**a**) MF and (**b**) UF membranes filtrated with TA after soaking in NaOH solution. Fitting performed by exponential equations. S.E. stands for standard error.

Figure 6 shows that the tensile strength data were well-fitted to the exponential model (i.e., y(t) = y_0_ + ae^−bx^), resulting in an acceptable R-squared and standard error result. The results of the exponential model show that the UF membrane may age 1.8 years after filtration with tannic acid solution and cleaning with NaOH when the tensile strength decreases to 10% against the original membrane, and seven years when the tensile strength decreases up to 30% (Table 3). On the other hand, the ageing of MF membrane was estimated to be about 5.1 years when the tensile strength decreased by 10%. The ageing of MF would be longer than 5.1 years if a tensile drop of more than 10% is allowed (i.e., 30% of the initial tensile strength, Table 3), based on the equation indicated in Figure 6. This estimation is consistent with a recent finding in full-scale water treatment plants where the mechanical properties of PVDF deteriorated and filtration performance dropped after 5 years of operation [13].

## 4. Conclusions

The following conclusions can be drawn from this study:

- Both UF and MF membranes were able to completely retain suspended solids, yet retain small amounts of tannic acid. The UF membrane was able to retain more TOC than the MF membrane. Tannic acid molecules adsorb onto the membrane surface, which results in changes in surface characteristics, especially surface functional groups that are responsible for enhancing membrane’s hydrophilicity.
- NaOH could remove tannic acid and citric acid could remove inorganic matter (Fe_2_O_3_) that fouled the membranes. However, it was confirmed that NaOH had a stronger effect on the tensile strength and surface chemistry of the fouled MF and UF membranes than citric acid. The results infer the relationship between the fouling effects and changes in surface functional groups associated with chemical cleaning.
- Prediction of lifetime by an exponential (decay) model confirmed that the UF membrane cleaned with NaOH would be aged within about 1.8 years and the MF membrane after about 5 years, when a 10% tensile strength decrease against the original membrane is allowed (cleaning every 15 days, downtime 2 h per cleaning).

## Figures and Tables

**Table 1:** Characteristics of the synthetic cashew nut processed waters.

Parameter	Water A	Water B
TSS ^a^ (mg/L)	240	240–250
TDS ^b^ (mg/L)	3200	3200
COD ^c^ (mg/L)	4000	4000
Tannic acid (mg/L)	100	100
Iron oxide	-	10 mg/L as Fe_2_O_3_
pH (buffered)	7.50	7.50

Note: ^a^ Total suspended solid (TSS) adjusted by adding Kaolin; ^b^ Total dissolved solid (TDS) adjusted by adding NaCl; ^c^ Chemical oxygen demand (COD) adjusted by adding glucose.

**Table 2:** Tensile strength, modulus of elasticity, and elongation of virgin and NaOH-cleaned MF membranes.

Hollow-Fiber MF Membrane	Before Hydrolysis (Virgin Membrane)	After Hydrolysis (Soaking in NaOH 2.5 wt.% for 2 Weeks)
Tensile strength (MPa)	6.11 ± 0.2	5.46 ± 0.23
Elongation (%)	50.80 ± 3.4	39.2 ± 7.2

**Table 3:** Membrane ageing predicted by the data obtained from cleaning with NaOH solution after filtration with TA solution using the exponential models presented in Figure 6.

Tensile Strength Decay (% of the Initial Value)	Predicted Lifetime (Years)	
	MF	UF
10	5.1	1.8
30	>5.1	7

**Figure A9:**
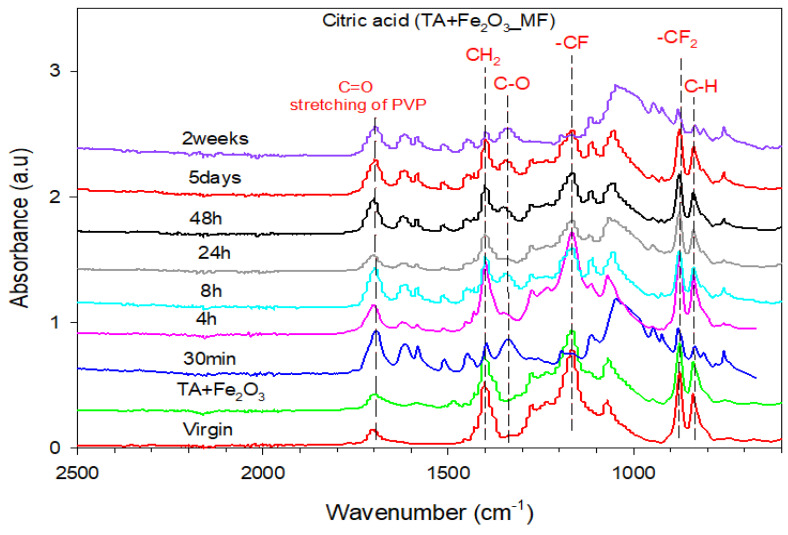
FTIR spectra of PVDF MF virgin and fouled membranes of Tannic Acid with iron oxide solution after soaking in Citric Acid at different accumulative soaking time.

## Data Availability

Not applicable.

## Appendix A

**Table A1:** Measured properties of the MF and UF membranes.

Items	MF membrane	UF membrane
Manufacturer	Kuraray Asia Pacific Ltd.	NANOTEC
Material	PVDF	PVDF (19%)
Configuration	Hollow-fiber	Hollow-fiber
Fiber diameters (mm)	O.D. 1.4 mm/I.D. 0.7 mm	O.D. 1.76 mm/I.D. 0.85 mm
Hydrophilic Additive (HA)	PVP	PEG
Pore size	0.02 µm	100–200 kDa
Contact angle (°)	61.6	85.4
Tensile strength (MPa)	6.11	5.35
Young modulus (Mpa)	200.9	153.8

Note: PVDF (Polyvinylidene fluoride), PVP (Polyvinylpyrrolidone), PEG (Polyethylene glycol).

**Table A2:** Permeate water quality of microfiltration.

		Turbidity (NTU)	TSS (mgl^−1^)	TOC (mgl^−1^)	pH (-)	Temp. (°C)
Tannic acid wastewater	Feed water	208.3±19	271 ± 102	1387 ± 13.4	7.5 ± 0.1	25.3 ± 1.0
	Permeate water	0.25 ± 0.04	N/A	1383 ± 2.8	7.4 ± 0.18	25 ± 0.9
Fe_2_O_3_ with Tannic acid wastewater	Feed water	230.24 ± 72.45	N/A	1464 ± 94.36	7.42 ± 0.07	25.68 ± 0.64
	Permeate water	0.208 ± 0.15	N/A	1380 ± 126	7.23 ± 0.1	25 ± 0.1

**Table A3:** Permeate water quality of ultrafiltration.

		Turbidity (NTU)	TOC (mgl^−1^)	pH (-)	Temp. (°C)
Tannic acid wastewater	Feed water	145.4 ±45.69	1387 ± 13.4	7.28 ± 0.06	29
	Permeate water	0.30 ± 0.09	1383 ± 2.8	7.25 ± 0.04	29
Fe_2_O_3_ with Tannic acid wastewater	Feed water	161.41 ± 36.28	1464 ± 94.36	7.3 ± 0.005	29
	Permeate water	0.34 ± 0.11	1380 ± 126	7.23 ± 0.041	29
